# Communication Skills and Professional Practice: Does It Increase Self-Efficacy in Nurses?

**DOI:** 10.3389/fpsyg.2020.01169

**Published:** 2020-06-12

**Authors:** César Leal-Costa, Sonia Tirado González, Antonio Jesús Ramos-Morcillo, María Ruzafa-Martínez, José Luis Díaz Agea, Carlos Javier van-der Hofstadt Román

**Affiliations:** ^1^Nursing Department, University of Murcia (UM), Murcia, Spain; ^2^Instituto de Investigación Sanitaria y Biomédica de Alicante (ISABIAL), Alicante, Spain; ^3^Health Psychology Department, Miguel Hernandez University (UMH), Elche, Spain; ^4^Nursing Department, Catholic University of Murcia (UCAM), Murcia, Spain; ^5^Hospital Psychology Unit, University Hospital of Alicante, Alicante, Spain

**Keywords:** communication skills, nursing professionals, self-efficacy, patient-focused model, professional practice

## Abstract

The relationship between nurses and their patients is changing significantly, resulting in a patient-focused model. This work aims to contribute new knowledge about the effect of communication skills on perceived self-efficacy of nursing professionals. A cross-sectional descriptive study was conducted with a final sample consisting of 242 nurses. Different instruments that measured communication skills and the general and specific self-efficacy of nursing professionals were utilized. A positive and statistically significant correlation (*p* < 0.01) between the communication skills and the perceived general and specific self-efficacy was obtained. Nursing professionals who have adequate communication skills feel more confident and more competent, fostering good interpersonal relationships with their patients, and therefore, their perceived general and specific self-efficacy improved as well. Teaching communication skills is important to help nurses cope with a broad range of stressors in their daily interactions with patients, increasing their perceived self-efficacy.

## Introduction

The relationship between nurses and their patients is currently undergoing significant changes, resulting in a model that is more patient-focused (Langberg et al., 2019). This new orientation stems from a holistic model, in which nurses and patients share responsibility and control, and is based on an egalitarian clinical relationship of balanced power and control (Street, 2017; Treiman et al., 2017). Among all health care professionals, nurses spend the most time with the patients; therefore, having and using appropriate communication skills with their patients is highly important and needed (Pehrson et al., 2016). According to previous studies, good communication skills of health professionals are related to successful health outcomes, such as good patient satisfaction, adherence to treatment, and improvement of the indicators of quality of care (Beach et al., 2005; Stiefel et al., 2010; Uitterhoeve et al., 2010; Barth and Lannen, 2011; Lenzi et al., 2011; Bernard et al., 2012; Rezaei and Askari, 2014; Scholl et al., 2014; Capone, 2016). The most common problems related to the communication skills of the nurses reported by the patients were the lack of information about care, inability of tending to their emotional needs, and lack of respect (Nørgaard et al., 2012). These problems thus highlight the need to investigate the communication skills of the nurses and thus their self-efficacy when performing their professional functions.

The self-efficacy construct refers to a person’s own estimation of his or her ability to perform a specific task successfully (Bandura, 1978), and the perceived self-efficacy refers to the belief that the nursing professional has the skills needed to perform the necessary actions to obtain the desired results at work. Previous studies have investigated the self-efficacy of nurses and other health professionals in training sessions related to communication skills. These programs were effective, and the professionals increased their self-efficacy post-training in diverse relational contexts (Parle et al., 1997; Ammentorp et al., 2007; Doyle et al., 2011; Nørgaard et al., 2012). Nevertheless, there is an information gap about how the communication skills (provide and obtain information, respect, empathy, etc.) of the nurses relate to their self-efficacy when performing nursing interventions.

Thus, this work aims to contribute new knowledge about the effect of communication skills (informative communication, empathy, respect, and social skill) on perceived self-efficacy (general and specific with relational skills) of nursing professionals.

In this study, the following hypothesis was tested:

H1: Communication skills are positively associated with general and specific self-efficacy of nursing professionals.

## Method

### Design

A cross-sectional correlational *ex post facto* study conducted with a nursing professionals’ sample from Alicante, Spain.

### Participants

The sample was selected through non-probability convenience sampling. As inclusion criteria, all the participants had to (1) work in hospital or primary care setting, (2) be a registered nurse, (3) be actively working with a minimum experience of 1 year, and (4) provide direct care to patients.

The sample size needed was calculated, and the results showed that a sample size of 242 nurses achieved 100% power to detect a difference of −0.50 between the null hypothesis correlation of 0.00 and the alternative hypothesis correlation of 0.50 using a two-sided hypothesis test with a significance level of 0.05 (Algina and Olejnik, 2003).

### Instruments

The participants completed a self-report questionnaire that included the following:

The participant’s sociodemographic and professional information, age, sex, marital status, other graduate and postgraduate degrees, length of time worked, type of center (public, public with private management, or private), and place of work (hospital, specialty center, primary care, or other), was evaluated.

The Communication Skills Scale (CSS; Leal-Costa et al., 2016, Leal-Costa et al., 2019) was utilized to evaluate the communication skills of the nurses. This scale is composed of 18 items, with a Likert-type response scale, and was scored with a 6-point frequency scale which indicated how often they performed the item. Two items were worded inversely (items 18 and 20). It included four dimensions: (a) Informative Communication, consisting of six items (5, 8, 9, 14, 17, and 18), which reflect the manner in which the health professionals obtain and provide information on the clinical relationship they establish with patients; (b) Empathy, composed of five items (2, 4, 6, 11, and 12), which reflect the capacity of the health professionals to comprehend the feelings of patients and make their empathy evident in the relationship, as well as the behavioral dimension, the empathic attitude, composed of active listening and empathic response; (c) Respect, with three items (21, 3, and 15), which evaluate the respect that is shown by the health professionals in the clinical relationship established with patients; and d) Social Skills, with four items (17, 10, 13, and 16) that reflect the ability of the health professionals to be assertive or to exhibit socially skillful behaviors in the clinical relationship established with patients. The Cronbach’s alpha (α) was 0.77 for empathy, 0.78 for informative communication, 0.74 for respect, and 0.65 for social skills. Evidences of content validity were obtained with a qualitative evaluation phase of the items with a group of experts and construct validity which related the communication skills with burnout (Leal-Costa et al., 2016).

### General Self-Efficacy Scale

The Spanish adaptation of Sanjuán Suárez et al. (2000) scale was used, which consists of 10 items with a 4-point Likert response scale. It measures the level of general self-efficacy, understood as a global construct that refers to the stable belief of people about their ability to adequately handle a wide range of stressors from everyday life. The scale had a Cronbach’s α of 0.87 and 0.88 correlation halves.

### Specific Self-Efficacy Scale

The Specific Self-Efficacy Scale for communication in difficult situations (Doyle et al., 2011) is composed of two scales: the Extent of Difficulty Scale developed by Arranz et al. (2005) and the Confidence Scale developed by Parle et al. (1997). The difficulty scale measures the ability of nurses to handle problems during their interaction with patients, family, and the health care team. The “confidence” scale measures the confidence of the professionals themselves for handling situations regarding patients. The internal consistency for the self-efficacy scales was excellent (confidence = 0.88, difficulty = 0.90) (Doyle et al., 2011).

### Implementation

The data were collected from June to September 2016. The self-administered questionnaire with the tools described, informed consent, and project information sheet were created with the Google Forms tool. Collaboration requests were distributed to all the nurses by the Nursing Council of the Province of Alicante. The online questionnaires were sent *via* e-mail on two separate occasions to obtain the sample size needed and to ensure its representativeness. All the response data from the nurses were collected in a Google spreadsheet for analysis.

### Data Analysis

Data analysis was performed using the SPSS statistical package version 22.0 (IBM Inc. 2013, NYC) and AMOS version 18.

Descriptive statistics were calculated as frequencies for categorical variables, whereas means and standard deviations were computed for continuous variables. For the analysis of the correlation between the communication skills and self-efficacy, Pearson’s bivariate correlations were used.

The variables showed adequate normality for the maximum likelihood estimation (MLE) method, i.e., skewness >2–3 and kurtosis >7–10 (Marôco, 2014). To test the model hypothesis, a path analysis technique was performed. The significance of the regression coefficients was evaluated after the estimation of the parameters with the maximum likelihood estimation method. The significance of effects was assessed with bootstrap resampling (Marôco, 2014). The effects with *p* ≤ 0.05 were considered significant. The fit of the model was evaluated using the χ^2^/*df* < 5; the root mean square error of approximation (RMSEA) values ≤0.08, and the comparative fit index (CFI), goodness of fit index (GFI), and Tucker–Lewis (TLI) index values ≥0.90 indicate a good fit (Kline, 2011).

### Ethical Considerations

All the participants gave their consent to participate in the study, which was approved by the Ethics Committee of the University Miguel Hernández, Spain (reference number DPS-CVR-001-11). The principal bioethical aspects were settled by ensuring voluntary and informed participation and the confidentiality of the data and information of the study participants.

## Results

The sample was composed by 242 nurses, of whom 207 (85.5%) were women and 35 (14.5%) were men. The participants’ average age was 39.60 years [standard deviation (SD) = 10.51], and the average time working as a nurse was 16.62 years (SD = 21.09). Regarding their civil status, 53 (21.9%) were single, 167 (69%) were married or cohabiting, 15 (6.2%) were separated, and seven (2.9%) were widowers. The distribution according to type of management of the centers and place of work showed that 182 (75.2%) worked in public centers, 31 (12.8%) in privately run public centers, and 29 (12%) in private centers. Furthermore, 153 (63.2%) worked in hospitals, 41 (16.9%) in primary care, four (1.7%) in specialty centers, and 44 (18.2%) in other areas, such as nursing homes, municipalities, and non-hospital emergency centers. When asked if they had another bachelor’s degree (i.e., other than nursing), 38 participants (15.7%) said yes; these included psychology, anthropology, physiotherapy, nutrition and dietetics, and social work. As to whether they had a postgraduate degree, 106 (43.8%) answered yes; these were in nursing-related disciplines.

Regarding the reliability analysis of the scales, the internal consistency (α) of the CSS was 0.92 for the complete scale, 0.79 for empathy, 0.80 for informative communication, 0.77 for respect, and 0.70 for social skill. The α of the General and Specific Self-Efficacy Scale was 0.87 and 0.93, respectively.

The results showed that the nursing professionals had good communication skills and perceived self-efficacy (general and specific with nursing communication) (Table 1).

**TABLE 1:** Descriptive statistics of the Communication Skills Scale and the General and Specific Self-Efficacy.

**Instruments**	***M***	**SD**	**Skewness**	**Kurtosis**
Communication skills scale	89.26	9.89	–0.14	–0.79
Empathy	24.77	3.36	–0.37	–0.51
Informative communication	30.91	3.44	–0.27	–0.87
Respect	15.73	2.02	–0.59	–0.70
Social Skills	17.85	3.13	–0.29	0.73
General self-Efficacy scale	32.36	4.25	–0.40	0.50
Specific self-Efficacy scale difficulty	86.26	11.90	–0.97	1.39
Specific self-Efficacy scale confidence	64.12	12.63	–0.77	0.94

As for the relationship between communication skills and self-efficacy, a positive and statistically significant correlation was observed (*p* < *0*.01) (Table 2). Correlation coefficients were obtained that oscillated between 0.265 and 0.606, with the strength of the relationship being moderate.

**TABLE 2:** Bivariate correlations between the total and dimensions of the CSS and the General and Specific Self-Efficacy.

**CSS**	**GSS**	**SSSD**	**SSSC**
Total	0.493**	0.400**	0.578**
Empathy	0.464**	0.384**	0.606**
Informative communication	0.433**	0.329**	0.478**
Respect	0.334**	0.265**	0.367**
Social Skill	0.367**	0.320**	0.413**

**p < 0.01. CSS, Communication Skills Scale; GSS, General Self-Efficacy Scale; SSSD, Specific Self-Efficacy Scale Difficulty; SSSC, Specific Self-Efficacy Scale Confidence.

**FIGURE 1:**
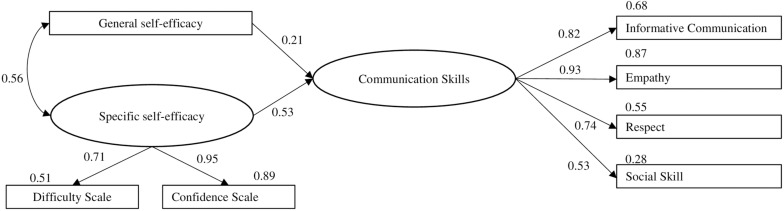
Model of the effect of communication skills and general and specific self-efficacy of nursing professionals with standardized parameter estimates.

The proposed model (Figure 1) attempted to test the hypothesis of the relationship between communication skills and general and specific self-efficacy of nursing professionals. Figure 1 shows the model with the standardized regression coefficients and *R*^2^ values.

The model fit showed a moderate adjusted model χ^2^/*df* = 3.22, RMSEA = 0.09 (90% CI = 0.063−0.131), CFI = 0.97, GFI = 0.96, TLI = 0.94. All the trajectories were statistically significant (*p* < 0.05).

## Discussion

Nursing professionals can experience difficulties when communicating with patients, patients’ family, and other colleagues in the clinical context (Arranz et al., 2005). However, having good communication skills can be important to help nurses cope with a broad range of stressors in their interactions with patients, family, and other colleagues, increasing their perceived self-efficacy. This study aimed to analyze the relationship between communication skills and general and specific self-efficacy of nursing professionals.

The results showed that the nursing professionals had high scores in the dimensions of communication skills and self-efficacy (general and specific with nursing communication). These results are consistent with another study conducted with a larger sample of health professionals (doctors, nursing professionals, and auxiliary nursing) (Leal-Costa et al., 2015, 2019). In the multivariate analysis, we found the expected effect of the communication skills on general and specific self-efficacy, confirming the proposed hypothesis.

The results obtained also provided a model which indicated that if nurses have good communication skills, these can contribute to making them feel more secure and competent and can thus enhance their interpersonal relationships with the patients. This in turn helps nurses to cope with a broad range of stressors, thus increasing their self-efficacy (general and specific to nursing communication). These results were consistent with other similar studies (Parle et al., 1997; Arranz et al., 2005; Ammentorp et al., 2007; Doyle et al., 2011; Nørgaard et al., 2012; Park et al., 2015; Chan and Sy, 2016).

Thus, nurses who have good communication skills could feel more secure and competent in their interactions with patients, indicating that teaching communication skills is important to help nurses cope with a broad range of stressors and to increase their perceived self-efficacy. The adjusted model used in this study demonstrated the relationship between communication skills and general and specific self-efficacy.

Another study (Park et al., 2015) found a positive correlation between communication skills and perceived self-efficacy of nurses of an emergency department. However, our work establishes a model of the effect of the communication skills on general and specific self-efficacy, with standardized regression coefficients of 0.21 and 0.53, respectively.

Therefore, increasing the communication skills of the nurses can positively affect the interventions performed in the different care services, as the nurses feel able to perform the actions needed to obtain the results sought in their work.

### Limitations

The study had several limitations. On the one hand, the study was carried out in a single province, with nursing professionals working in primary and specialized care. A study which includes more provinces would enrich the results and could reinforce or weaken the results obtained. Also, the sample was selected using a non-probabilistic convenience sampling method, and the study was cross-sectional. Thus, the generalization of the results should be made with caution, with studies that use more complex designs needed in order to show how the communication skills of the nurses affect their perceived general self-efficacy. To show the existence of a causal relationship, experimental designs should be implemented with random assignment of the subjects to the study conditions.

## Conclusion

Despite the above limitations, we can establish the following conclusion: our findings support the hypothesis that communication skills can positively affect perceived self-efficacy (general and specific with relational skills).

## Data Availability Statement

The datasets analyzed in this article are not publicly available. Requests to access the datasets should be directed to cleal@um.es.

## Ethics Statement

All the participants gave their consent to participate in the study, which was approved by the Ethics Committee of the University Miguel Hernández, Spain (reference number DPS-CVR-001-11). The principal bioethical aspects were settled by ensuring voluntary and informed participation and the confidentiality of the data and information of the study participants.

## Author Contributions

CL-C, JD, AR-M, ST, MR-M, and CH contributed to the conceptualization, formal analysis, and writing, reviewing, and editing. JD, ST, and CL-C contributed to data curation. JD, AR-M, MR-M, and CL-C contributed to the investigation. JD, CH, MR-M, ST, and CL-C contributed to the methodology. ST and CL-C and contributed to project administration. CH and CL-C contributed to the resources. AR-M and CL-C acquired the software. CH and CL-C contributed to supervision. CL-C, ST, and MR-M contributed to validation. CL-C, ST, and CH contributed to writing the original draft.

## Conflict of Interest

The authors declare that the research was conducted in the absence of any commercial or financial relationships that could be construed as a potential conflict of interest.
